# Book Review

**Published:** 1897-12

**Authors:** 


**Medicine and Kindred Arts in the Plays of Shakespeare.** By Dr. JOHN MOYES. Pp. xiv., 123. Glasgow: James MacLehose & Sons. 1896.—A melancholy interest attaches to this work, which has been published posthumously. The somewhat imperfect manuscript left by the author was prepared for the press by Dr. J. Finlayson, who provided also a preface and a limited bibliography of the subject, which includes the presidential address delivered to the Bristol Medico-Chirurgical Society in 1887. Dr. Moyes originally presented for his graduation thesis the foundation of the book, and afterwards improved and enlarged it, receiving some aid both from his subsequent editor and from Brinsley Nicholson. Unable to gain more than occasional access to a large library, and unwilling to read any of the other literature upon this topic, Dr. Moyes industriously compiled all the passages in Shakspere bearing on medicine, and elucidated them with comment and explanation. He evidently was not very closely acquainted with recent Shaksperian criticism, and took for granted that the poet wrote every line which is found in the generally-accepted canon of his works, and invented the incidents of the plays. In the book in its present form it is not possible to determine with precision what is attributable to Dr. Moyes, Dr. Finlayson, or to Dr. Brinsley Nicholson respectively, but whereas most of the allusions are briefly commented upon, some are dilated upon with fuller reference and greater detail. While decidedly an interesting book, and giving proof of much labour and study of the text upon the deceased author's part, it can not be considered as a matured discussion of the subject. To properly explain the many allusions to the medical art by Shakspere demands a full knowledge of the views on anatomy, physiology, and therapeutics which obtained in England during the latter half of the 16th century, as well as the opportunity of consulting many books only to be found in libraries of the first class. Without disrespect to Dr. J. C. Bucknill, our present author, or others, we may say that *the* book on medicine in Shakspere has yet to be written. This work is attractively printed, and a word of praise must be given to the index, although this might have been made still more useful if an index of the quoted passages had been given.

**Notes on Medical Nursing.** By the late JAMES ANDERSON, M.D. Edited by ETHEL F. LAMPORT. Third Edition. Pp. xiv., 184. London: H. K. Lewis. 1897.—This is almost a *verbatim* reprint of the second edition, upon which we commented at the time of its appearance two years ago, and need not therefore offer any general criticism on this occasion. There has been added to this edition a small glossary of the more technical terms used in the text. In the preface it is stated that this is done in compliance with request by a large number of readers, which "shows that this little book has reached beyond the purely professional circle, and is a most gratifying testimony to its usefulness!" We question whether such a book in the hands of the laity could lead to anything but confusion.

**Antiseptic Principles for Nurses.** By C. E. RICHMOND. Pp. 47. London: J. & A. Churchill. 1897.—This booklet is written in popular style, intended to teach nurses the theory of asepsis and antisepsis. It assumes that nurses "know the different substances used, and observe the great precautions adopted in connection with the dressings of operation wounds." Three chapters describe: (1) Wounds: healthy and unhealthy. (2) Germs: their nature, growth, development, and action. (3) Asepsis and antisepsis. It is questionable whether it is desirable to illustrate and describe micrococci, bacilli, diplococci, multiplication by spores and fission, &c., to those unacquainted with microscopy. Moreover, no instructions are given telling a nurse how to scrub her hands and clean her finger-nails, to avoid changing a napkin before doing a dressing or blowing her nose when she is handing sponges. The book is readable from the theoretical standpoint; only, it does not show nurses how to wash and be clean.

**The Matron's Course.** By Miss S. E. ORME. Pp. 87. London: The Scientific Press, Limited. 1897.—The probationer or person beginning to learn the art of nursing will find much useful matter in this little book. The advice is wholesome, clearly and graphically expressed in the form of short lectures. The book will repay careful perusal; and though the beginner may find some scientific terms unexplained, care has evidently been taken to use homely and well-known words wherever possible.

**Saint Thomas's Hospital Reports.** New Series. Vol. XXIV. London: J. & A. Churchill. 1897.—This volume opens with an exhaustive account of pulmonary hypertrophic osteo-arthropathy by Dr. F. R. Walters, together with abstracts of sixty-five published cases, which will be of value to those who are working at this still somewhat obscure subject. There are photographs and skiagrams of the hands of a patient suffering from this affection who came under Dr. Walters's own observation, and whose case is reported in full in a separate paper. A reprinted paper by the late Mr. Geo. Rainey is of interest in connection with the artificial production of organic forms. Mr. Makins reports three instructive cases of injury to the abdominal viscera, one of them remarkable for the complication of disorders from which the patient suffered. Mr. Clutton contributes a paper on the treatment of fracture of the patella by wire suture through an open wound, with a number of successful cases in his practice, and Mr. H. B. Robinson one on the rarer forms of tubercular joint disease. Amongst other articles of interest is a good practical paper by Dr. F. Foord Caiger on the occurrence of relapse in the specific fevers. He states that in scarlet fever a relapse occurs in from five to six per cent. of cases treated in a fever hospital, and considers that it is more frequent in hospital than in private practice, and of more common occurrence than is generally held by the medical profession; the cause is probably re-infection from without. There are the customary reports of the work done at the Hospital during the past year, and we would call attention to the valuable abstracts of the more important and interesting cases. We note that the enteric fever mortality was 12.5 per cent. The results of the antitoxin treatment in diphtheria are as follows: average mortality of all cases for ten years previous to use of antitoxin, 49.8 per cent.; for 1895, during which antitoxin was used, 25.4. In 1894, prior to use of antitoxin, there were 63.79 per cent. laryngeal cases; in 1895, 37.25 per cent.: of these cases the mortality was 66.03 per cent. in 1894, 37.25 per cent. in 1895, and tracheotomy was required in 91.37 per cent. and 87.93 per cent. of them in 1894 and 1895 respectively. This diminution of mortality was accompanied by a considerable increase in complications; *e.g.* there was albuminuria in 28.3 per cent. in 1894, and in 47.27 per cent. in 1895. A table on p. 243 brings out the importance of early treatment. Sufficient has been said to show that this volume is of much value and interest, and we commend it to our readers.

**Transactions of the Iowa State Medical Society.** Volume XIV. Des Moines: The Kenyon Printing and Manufacturing Company. 1896.—This society shows considerable signs of vigour in its forty-fifth year. It brings together a large number of papers on subjects of importance, and prints them in a well got up volume. We find Shrader and others writing on ovarian neuroses, Leipziger and Cokenower on empyema, while the lively and fully reported discussions on the papers bring forward useful and sometimes amusing criticisms. The volume is an interesting one, and we are much impressed with the amount and quality of the work done by the members of a society in a single State of the Great Union.

**Transactions of the American Surgical Association.** Vol. XIV. Philadelphia: Printed for the Association. 1896.—The present volume contains a number of papers of great value, although the topics of which they treat are scarcely so attractive as some which have appeared in previous volumes. Such topics as "The Surgical Treatment of Tuberculosis," "The Ambulatory Treatment of Fractures," "The Surgical Peculiarities of the Negro," &c., are ably dealt with, and further illustrated by a report of the discussions which followed the reading of the papers. It is evident that the Fellows of the American Surgical Association maintain their reputation of being in the front rank of modern surgery.

**Transactions of the Ophthalmological Society of the United Kingdom.** Vol. XVI. London: J. & A. Churchill. 1896.—Though not containing much that is very new, this volume more than maintains the high standard of those which have preceded it. Professor Snellen, in his "Bowman Lecture," records some important observations on the relationship of acuity of vision to intensity of illumination. There are records of a great number of interesting cases, which are well illustrated, and accurately, and what is more, tersely described. Dr. George Bull's paper on "The Visual Effects of Refractive Error" supplies an answer to much that has been written on so called "monocular diplopia." The work of Mr. George Dean and Mr. C. H. Usher at Aberdeen is very interesting and important. By section of the retinal nerve fibres in rabbits they show that degeneration occurs in the corresponding quadrant of the optic nerve through its entire length; also that, in the monkey, the degeneration caused by a lesion between the macula and the disc coincides in position with the degeneration found by Nettleship and others in cases of toxic amblyopia. From the statistics of one hundred cases of intrao-cular tumour, Mr. Devereux Marshall concludes that the increase of tension, which was present in 53 per cent. of the eyes examined, is due to diminution of the angle of the anterior chamber, and that a sarcoma in the posterior half of the eye is the tumour most likely to cause glaucoma. Mr. Cross, in his paper on Hydrophthalmos, shows that the disease is due to blocking of the angle between the cornea and the iris. This fact was demonstrated in sections of three eyes exhibited, and is well shown in illustrations reproduced from photomicrographs. In every case the canal of Schlemm was either absent or defective. The volume is excellently printed, and the subjects carefully arranged for reference.

**Transactions of the British Orthopædic Society.** Vol. I. Birmingham: Hall and English. 1896.—The British Orthopædic Society was founded in 1894, its preliminary meeting having been held in Bristol during the meeting of the British Medical Association. The present volume is the first published by the new society, and we hope it will be the precursor of a long and successful series. Interesting papers and discussions on various orthopædic subjects will be found fully reported and copiously illustrated.

**The Transactions of the Edinburgh Obstetrical Society.** Vols. XX. and XXI. Edinburgh: Oliver and Boyd. 1895 and 1896.—We regret that we have not space to notice in detail the interesting communications contained in these volumes. Dr. Fordyce's paper on "The Clinical Aspects of Utero-sacral Cellulitis" insists on the distinct symptoms and physical signs associated with this condition, which do not necessarily involve the presence of tubal or ovarian disease. Dr. Freeland Barbour points out the value of some recent frozen sections in advancing our knowledge of the mechanism of labour, and tabulates these results with great care. In a clinical note on the advantages of Walcher's position during delivery, Mr. Fothergill gives an interesting account of labour in cases of contracted pelvis wherein this position was used, and shows that an increase of the true conjugate up to half an inch can take place by its use. Dr. Buist's interesting paper on chorea must be read in the original to be properly appreciated. Dr. J. W. Ballantyne contributes an elaborate paper on Teratogenesis, a subject which he has made peculiarly his own, and which is worked out with his accustomed care and careful detail. An account of "Mittelschmerz," by Dr. Halliday Croom, is also interesting. He attributes the pain in this condition in those cases where there is no external manifestation to the fact that ovulation and menstruation are not simultaneous, and that the pain is caused by the difficult dehiscence of a follicle when the capsule of the ovary is thickened. In a paper on the "Indigestion of Breast Babies," Dr. James Carmichael draws attention to various conditions connected with the health of the mother which prejudicially affect the infant. He emphasises the necessity for examining the milk in all cases when a breast-fed infant shows signs of mal-nutrition or intestinal disturbance. Both volumes are well indexed, well printed, and contain many other valuable papers and discussions. An index to vols. i.—xxi. is added, which we owe to the industry of Dr. Ballantyne.

**Annual of the Universal Medical Sciences.** Edited by Charles E. Sajous, M.D. Five volumes. Philadelphia: The F. A. Davis Company. 1896.—This already voluminous work has grown larger. "To bring the usefulness of the Annual to its highest standard it was deemed advisable to increase the length of the abstracts." This has been done to the extent of over half a million words. The increase will naturally impose greater labour upon the physician who wishes to acquaint himself with the progress of the medical sciences as a whole, for we are informed that "the professional reader who seeks to familiarize himself with every branch of medicine can alone be considered as well informed nowadays. The epoch of absolute specialism belongs to the past; every disease known represents but one link of a chain, and to totally ignore portions of that chain is to refuse the light its other links may afford and to limit one's capabilities." It is clear that every practitioner, no matter how hard worked, must study these volumes; but the large editorial staff have provided an analytical index and cyclopædia of treatment, which gives in 327 pages "the active principle, as it were, of the whole year's labors." It is believed to be possible for an average reader to review the entire field of medicine and surgery, including the specialities, in a few evenings. We can but wonder how much of this skimming would be available for future use, as few readers have the capacity for condensed dictionary reading such as is implied. Many smaller items of improvement are mentioned, all adding much to the value of the work. We congratulate Dr. Sajous and his helpers on the completion of another instalment of this magnificent work. These volumes are dated 1896; clearly the work is too colossal to be done quickly, and its excellence must be considered as a sufficient explanation of the tardiness of its appearance. Notwithstanding the great increase in the cost of production, the extra matter presented, the analytical index, &c., the price of the work is not increased, and the editor hopes the publishers will have their reward in a more liberal patronage than heretofore.

**Injuries and Diseases of the Ear.** By MACLEOD YEARSLEY. Pp. 40. London: The Rebman Publishing Co., Ltd. 1897.—These papers, which have appeared before in the pages of *The Medical Times* and of *Pediatrics*, are now brought together in book form, and are well worthy of being read. They are distinguished by practical good sense, and contain many hints of value. It was a relative of Mr. Yearsley who first invented the cotton wool artificial drum-membrane, and one of the present papers deals in an interesting manner with this subject.

**The Swedish System of Physical Education.** By THEODORA JOHNSON. Pp. 79. Bristol: John Wright & Co. 1897.—Ling's Swedish system of physical exercises is now very largely adopted in schools and educational establishments all through this country, and Miss Johnson's book will prove of value to all engaged in educational work, as well as to the medical profession. It is mainly the reproduction of a paper read before the members of the British Medical Association at the annual meeting held in Bristol in 1894, and embodies the results of twelve years' experience in Bristol and of observations made in Sweden. It contains many useful descriptions of the exercises and movements best adapted for the treatment of spinal curvatures and other similar affections. The drawings were made by Dr. Theodore Fisher, and serve to illustrate the movements very faithfully.

**Practical Hints on District Nursing.** By AMY HUGHES. Pp. 99. London: The Scientific Press, Limited. 1897.—On the tact and care which should guide nurses in their work among the poor the writer of this little book is a competent authority. It should be in the hands and in the memory of every district nurse; for every page shows the experience of one who has worked among the poor, and points out little matters which if neglected render the work of a nurse useless and unacceptable. The nurse who takes to heart the useful advice here given will be saved much loss of time and annoyance. It must, however, be understood that this booklet is intended to supplement and not to replace treatises on nursing, otherwise the directions will often be insufficient, such as those on the care of the eyes in the newborn child. We doubt, too, the use of recommending carbolic oil for lice, whereas they and their offspring are easily destroyed by an application of carbolic lotion, if repeated after the eggs hatch, that is in fourteen days. The addition of an index would have been an advantage.

**Transactions of the Ohio State Medical Society.** Toledo: The Blade Printing and Paper Co. 1896.—There are several papers of interest in this volume, in addition to the information of local value. The president's address, by Dr. Dan Millikin, on " A Study in Credulity," is a stirring and masterly *résumé* of the work of medical men in the causes of science and discovery, and a severe denunciation of quackery in the profession. He instances the credulity of those who order castor, musk, valerian, and asafœtida, and calls iodoform the "druggiest of drugs," and says "the whole American profession have gone daft over these preparations of the manufacturing pharmacists;" and he has no words sufficiently scathing for the doctors who order preparations without even knowing the names or the doses of the ingredients composing them. He has also a useful and timely word of admonition concerning trashy journals, of which he does not hesitate to give the titles, and he justly condemns many of the best journals which often contain "no clean advertisements, such as should accost the physician, with the exception of, here and there, a call to drink pale ale, to buy trusses or artificial legs, or to go to a private lunatic asylum." Dr. J. E. Pilcher ably defends vivisection in the cause of true humanity. There are also papers on recent studies in gastric digestion, by Dr. J. T. Whittaker; on intestinal obstruction, by Dr. M. Stamm; on four successful cases of total extirpation of the larynx, by Dr. G. W. Crile; on perforating ulcer of the stomach, by Dr. W. J. Gillette; on anchoring the kidney, by Dr. R. H. Reed; several papers on diseases of the eye, on middle ear disease and on gonorrhœa, and a paper by Dr. J. F. Baldwin on abdominal hysterectomy. Several of the articles, particularly those mentioned above, are well worth perusal.

**Archives of the Roentgen Ray.** London: The Rebman Publishing Company, Limited.—Under this title the periodical which made its first appearance last year as *The Archives of Skiagraphy* commenced its second volume in July. Its change of name is a consequence of a change in its scope and purpose. Instead of being as heretofore almost entirely a portfolio of skiagrams, with a few explanatory notes, it is in future to be virtually the organ of the recently formed "Roentgen Society," which has as its aim the study of the rays associated with the discoverer whose name it bears, in all their various aspects and applications—physical, medical and commercial. The reproduction in its pages of skiagrams of special interest is still a prominent and valuable feature. Photographs of this kind are peculiarly difficult to reproduce satisfactorily by any of the "processes" adapted for book illustration, but the examples included in the number of the *Archives* before us must be regarded as very successful. They include, among others, a skiagram of an entire adult taken by a single exposure; one of the chest of a young child suffering from phthisis; and a third giving clear proof of the presence of stone in the bladder. In addition, however, to these plates and brief descriptions of them there are ten pages of letterpress. Besides a collection of "Notes" from various sources and an account of the proceedings of the first meeting of the Roentgen Society, there is a contribution from Prof. Silvanus Thompson (the President of the Society) in the shape of an excellent epitome of the chief theories which up to the present have been advanced to explain the nature and properties of the X rays. Dr. W. S. Hedley follows with "a survey" of the subject, "present and retrospective," in which a summary of the applications and achievements of skiagraphy finds a prominent place. If we may refer to one item in his list of its medical accomplishments, surely Dr. Hedley does not adequately represent the degree of success already attained in applying skiagraphy to the diagnosis of renal calculus. Judging from local experience of the method, since the presence of stone in the kidney had been shown in at least two cases[1] photographed in the Skiagraphical Laboratory at University College, Bristol, prior to the appearance of Dr. Hedley's article, it would seem that skiagraphy had at the time shown considerably greater promise of successful application to such cases than his account of it would suggest. But, to return to the publication itself, we can heartily congratulate the Editors upon the change which the *Archives* have undergone. In their present form, dealing as they do with their subject from a physical standpoint as well as from a medical one, they are sure to excite very much wider interest, and are likely to be instrumental in greatly assisting the advance of the subject, both as regards its theoretical elucidation and the development of its practical side.

**Sixty-second Annual Report of the British Medical Benevolent Fund, for the Year 1896.** London: Morton & Burt.—In this Report the Committee say that, in respect of the Grants Fund, they regret "that it is still far too small to meet the pressing wants of the many candidates for help. The number of applicants

[1] For an account of one of these, see an article by Dr. James Swain, *Bristol Medico-Chirurgical Journal*, March, 1897, p. 9.

continues to be very large, and the cases most deserving—many of them very distressing, while both the donations and annual subscriptions have fallen far short of the requirements." The amount contributed from the Bristol District is much less than it should be to a charity which appeals so directly to medical men. The Local Hon. Sec. is Dr. J. Michell Clarke, who will, we feel sure, be glad to supply a copy of the Report to anyone who desires, and to receive additional subscriptions.

